# Identification of Marker Immunoglobulin Rearrangements for Use by HAT-PCR in Myeloma

**DOI:** 10.3390/ijms27167128

**Published:** 2026-08-09

**Authors:** Elizabeth Hughes, Alexander Morley

**Affiliations:** College of Medicine and Public Health, Flinders University, Adelaide, SA 5042, Australia

**Keywords:** myeloma, residual disease, MRD, HAT-PCR, PCR, NGS, immunoglobulin gene, hypermutation

## Abstract

Libraries for next-generation sequencing were prepared using primers directed to framework 2, framework 3 and D and the 6 J regions of the rearranged immunoglobulin gene from 50 diagnosis myeloma bone marrow samples. After sequencing and bioinformatic analysis, a marker sequence suitable for minimal residual disease analysis by HAT-PCR was obtained for 90% of the 50 samples studied. In 88% of samples a complete VDJ sequence was obtained. Failure to detect a marker sequence was principally due to a low percentage of plasma cells in the marrow sample. Evaluation of 24 pairs of primers showed that they were highly specific and, when used in HAT-PCR, they provided accurate quantification. The high frequency of detection of a suitable marker sequence indicates that minimal residual disease can be monitored by HAT-PCR in the great majority of instances.

## 1. Introduction

Quantification of minimal residual disease (MRD) is now routinely used in clinical trials of treatment for myeloma as it is a powerful predictor of response and survival [1]. Although quantification of MRD is not routinely used for individual patient management, the emerging data from clinical trials strongly suggest that it will soon be used for this purpose [2,3,4]. If so, when deciding which method to use for measuring MRD, practical issues such as availability and cost will become important.

Until the present time the two methods most used for quantification of MRD in myeloma have been multiparameter flow cytometry (MFC) and next-generation sequencing (NGS). These methods have sensitivities of 10^−5^ and 10^−6^, respectively [5,6,7]. Recently we have described a modification of PCR, termed HAT-PCR (**H**igh **A**/**T** or **H**igh **A**nnealing **T**emperature-PCR) [8]. The essential features of HAT-PCR are 1–3 A or T bases at the 3′ end of the primer and an annealing temperature which is very close to the maximum possible temperature while still maintaining amplification efficiency. These two features markedly increase the specificity of the PCR, which not only reduces the frequency of false positives but also reduces the silent amplification of non-target genomic sequences which otherwise frequently brings amplification to a halt [9] and prevents the achievement of high sensitivity. As a result of these modifications, HAT-PCR has shown a sensitivity of 10^−6^ in studies of acute lymphoblastic leukaemia, chronic lymphocytic leukaemia and myeloma and, in direct comparisons with MFC and NGS, has been shown to be more sensitive than MFC and to have the same sensitivity as NGS [8,10,11].

HAT-PCR requires that the marker sequence of the rearranged *immunoglobulin* gene is known and this is conveniently determined using NGS. In this manuscript we describe our methodology for preparing the libraries for sequencing, the problems encountered and the results obtained after sequencing and bioinformatic analysis.

## 2. Results

The results are shown in Table 1. A marker usable as a target for PCR quantification of MRD was detected in 90% of the samples studied. In one sample a marker was detected only by the D primers but in 88% of samples a complete VDJ marker was obtained by the framework primers. A marker was detected in 40/50 samples by the Fr3-Fr3 protocol, in 37/50 by the Fr2-Fr3 protocol, in 35/50 by the Fr2-Fr2 protocol, in 35/50 by the Fr1-Fr2 protocol and in 22/50 by the D protocol. For the library most clearly identifying the marker, the sequence of the marker comprised >90% of the *immunoglobulin heavy chain* (*IGH*) rearrangement reads in 34 samples, 60–89% in seven samples and 40–59% in four samples.

The results in Table 1 are ranked to show the effect of plasma cell number. There was a significant association between a low number of plasma cells in the marrow aspirate and failure to detect a marker sequence by the framework protocols (Mann–Whitney U = 49, z = 2.49, *p* < 0.01). In the five samples in which plasma cells comprised ≤ 3%, a marker was not detected in three; a marker was only detected by D in one (38-26), and in one (20-26) the marker was not detected by the standard protocol but was detected when three replicates using a 250 ng of DNA were performed.

To examine the possibility that hypermutation may have interfered with marker detection, we examined the relation between the mutation frequency of the marker sequence and failure of detection of that sequence by the framework primers. Inspection of Table 1 shows that all four framework protocols successfully detected the marker sequence in 29 samples, but one protocol failed in six, two failed in five and three failed in four samples. Mutation frequency was slightly higher in the 15 samples showing failure of marker detection (median = 11.60%) than in the 29 samples not showing failure (median = 9.90%), but the difference was not significant (*p* = 0.457, Mann–Whitney U test).

The magnitude of non-specific amplification was assessed by calculating the Ct value for SYTO82 minus the Ct value for the IGH probe. For the four different framework protocols the mean value for Fr3-Fr3 was 1.78 (SE = 0.17, n = 99), for Fr2-Fr3 it was 0.03 (SE = 0.23, n = 52), for Fr2-Fr2 it was 0.07 (SE = 0.17, n = 75) and for Fr1-Fr2 it was 0.04 (SE = 0.13, n = 54). The mean value for the Fr3-Fr3 protocol was significantly greater (t test, *p* < 0.001) than for the three other framework protocols, each of which contained Fr2 primers.

**Figure 1:**
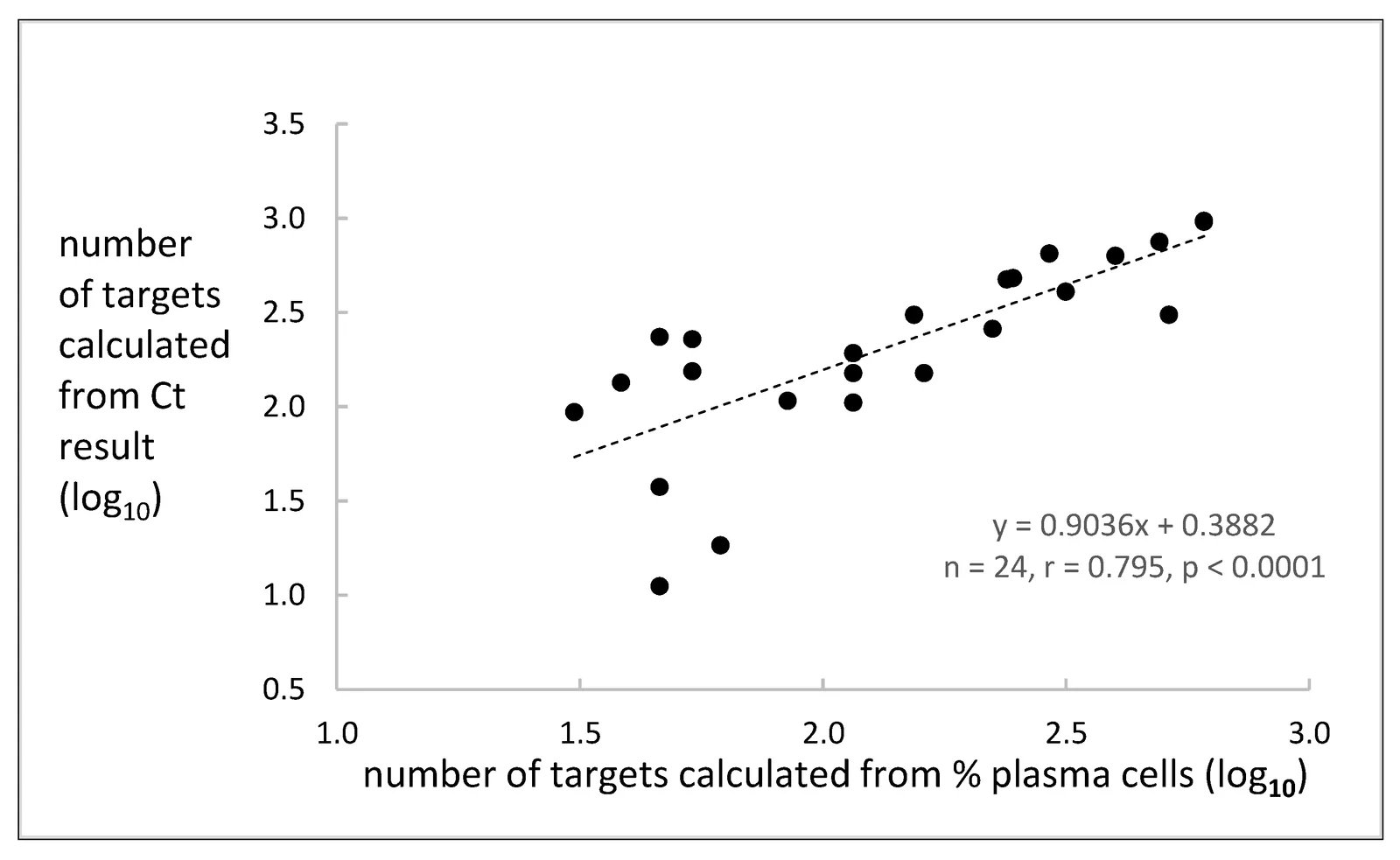
Relation between the number of targets in the PCR as calculated either from the percentage of plasma cells and mass of DNA or from the Ct result, the reported number of amplicons at threshold and the amplification efficiency of IGH primers.

A total of 24 primer pairs directed to identified marker *IGH* rearrangements were studied. When tested on 20 mcg of DNA there were no false-positive Ct results for 23 primer pairs and there was one false-positive result for one primer pair, at a level of 3.3 × 10^−7^. Figure 1 shows the relationship between the number of target rearrangements in the PCR either as determined from the percentage of plasma cells in the marrow or from the result of the PCR. The plasma cell percentage in different samples ranged from 5% to 79% and the close relationship over this range between the number of targets determined from the percentage of plasma cells and the number of targets determined by the PCR suggests both that quantification by PCR was accurate and that the identified marker rearrangements correctly marked the myeloma clone.

## 3. Discussion

Our results showed that a marker sequence can be detected by NGS in approximately 90% of cases of myeloma. In 88% of samples the marker sequence was a complete VDJ sequence and, as such sequences tend to be highly mutated in myeloma, they enable the synthesis of semi-specific reverse J primers. This improves the overall specificity of the PCR when quantifying MRD as was seen in our previous study of myeloma in which no false-positive PCR results were seen when 20 mcg from each of 32 patient samples was studied [10].

For myeloma, the identification of a marker *IGH* rearrangement by NGS faces two problems. Firstly, the focal nature of myeloma may result in a very low number of plasma cells being present within the marrow sample and, secondly, the presence of random mutations within the *IGH* gene interferes with hybridisation of the primers. A low number of plasma cells was found to significantly impair detection of a marker rearrangement but, in a minority of cases, this could be countered by performing the PCR in triplicate, using an increased amount of DNA, and pooling the results for analysis. Immuno-enrichment [12] may provide a way of dealing with this problem in the future.

To deal with the problem of hypermutation, we improved primer hybridisation by using primers at high concentration and performing hybridisation at a low temperature and for a prolonged time. The Jpool primers also contained multiple primers for each J region to allow for failure of binding due to hypermutation. The hybridisation of the primers to non-specific genomic sequences and the resultant amplification of non-specific material were countered either by performing two-stage semi-nested PCR or using “blockers” to interfere with non-specific amplification by the PCR. Non-specific amplification is monitored by SYTO82 and, as judged by the Ct difference between the IGH probe and SYTO82, non-specific amplification was increased in the protocols employing an Fr2 primer. This may have been a factor in the lower detection of markers by protocols containing an Fr2 primer.

We evaluated 24 primers which had been synthesised based on sequences derived by our method. They were tested by HAT-PCR and were found to be both specific and to provide accurate quantification. These results are in accord with our previous experience with HAT-PCR, and they emphasise the general utility of this method for quantification of MRD in neoplasms of lymphoid origin. Myeloma is a particularly good candidate as hypermutation affects the J regions as well as the V regions of the *IGH* gene and it is therefore possible to synthesise both a highly specific forward primer and a somewhat specific reverse primer for PCR. HAT-PCR now joins multiparameter flow cytometry and next-generation sequencing as potential methods for quantification of MRD. Each method has advantages and disadvantages, but the advantages of HAT-PCR are that it is very sensitive, simple, cheap, provides a rapid result, and is widely available.

## 4. Materials and Methods

### 4.1. Samples

Fifty anonymized diagnosis samples of bone marrow from patients with myeloma were obtained from the South Australian Research Cancer Biobank (SACRB). They had been donated with informed consent and the only information received with the samples was the percentage of plasma cells.

### 4.2. Preparation of Libraries

#### 4.2.1. Primers

Primers were synthesised by Integrated DNA Technologies (https://sg.idtdna.com/calc/analyzer, accessed on 1 August 2026). The Tm of the hybridising region of all primers was in the range of 70–76 °C except for that of the framework 2 (Fr2) primers for which it was 80–82 °C. The forward primers directed to framework 3 (Fr3), Fr2 and, framework 1 (Fr1) and the D region are shown in Table 2 and the reverse primers directed toward the J regions are shown in Table 3. As the primers comprised germline sequences and as the IGH rearrangements in myeloma may be heavily mutated, hybridisation of the primers to their target sequences may be suboptimal. To facilitate hybridisation, the Tm of the forward primers was quite high, they were used at high concentration and the PCR protocol involved annealing at a low temperature for a prolonged time. The conserved sequence in J, termed the probe sequence as it is used for binding of the probe in the PCR, was not available to be used as a reverse primer, Multiple downstream J primers were therefore used for each of the 6 J regions.

Although these features facilitated hybridisation of primers to their legitimate targets, they also facilitated non-target hybridisation of the primers and amplification of non-specific sequences. This could occur to such an extent that the PCR was inhibited. To minimise this problem, we used either semi-nested PCR, in which the second PCR round used internal forward primers, or the addition of blocking oligonucleotides to inhibit amplification of the non-target sequences. The latter approach used a BLAST search (https://blast.ncbi.nlm.nih.gov/Blast.cgi, accessed on 1 August 2026) to determine the sequence of an observed non-target amplicon and synthesis of oligonucleotides which could interfere with the amplification of that amplicon. The sequences of these blocking oligonucleotides and the J primers are shown in Table 3. For Fr3 amplification, the blocker was designed to hybridise strongly to the non-target amplicon and prevent polymerase extension; for Fr2 amplification, the blocker was designed similarly but was sited such that the 5′ six bases of the blocker hybridised to the same sequence as the 3′ six bases of the primer so that hybridisation of the primer was also inhibited; and for the amplification of the D rearrangement, the blockers were designed to prevent amplification of the full germline sequence between D7 and J1 by amplifying a small segment within it.

#### 4.2.2. PCR

Two rounds of PCR were used, the first round involving primers directed to conserved sequences in the rearranged *immunoglobulin* gene and the second round involving addition of the adapters and at times, the use of primers internal to those used in the first round. First round reactions were 10 µL and contained Platinum SuperFi II DNA Polymerase (Thermo Fisher Scientific, Waltham, MA, USA), (SF) (0.2 µL), MgCl_2_ at 5 mM, dNTPs at 300 nM, SF buffer and 50 ng of DNA. Second round reactions were 25 µL and contained Platinum Taq DNA Polymerase (Thermo Fisher Scientific), (PT) (0.4 µL), MgCl_2_ at 5 mM, dNTPs at 300 nM and PT buffer. A differently indexed reverse adaptor (RAD) primer was used for each sample.

Each sample was amplified twice, one sample with an IghJ probe at 160 nM and SYTO82 at 500 nM to assess specific and non-specific amplification, respectively, and the other sample as the library sample for NGS.

The individual constituents in all primer mixes and pools were equimolar. The concentrations refer to each individual constituent.

There were 5 samples in which the plasma cell percentage was ≤3%. For these, 3 duplicate PCRs were performed each containing either 250 ng of DNA for 2 samples or 500 ng DNA for 3 samples. The 3 sets of NGS data were pooled for analysis to determine the mean frequency of each rearrangement.

### 4.3. Fr3-Fr3 Protocol

1st round: Fr3ghi mix at 1000 nM, Jpool at 25 nM, 1A1bl3 at 50 nM, 1A2bl3 at 50 nM.

PCR protocol: 95 °C 1 min, 98 °C 1 min then 18 cycles of 95 °C 30 s, 50 °C 4 min, 70 °C 1 min.

2nd round: Fr3jkl mix at 50 nM, Individual RAD at 30 nM, 5 µL 1/20 diluted PCR product.

PCR protocol: 95 °C 3 min then 25 cycles of 95 °C 30 s, 70 °C 30 s.

### 4.4. Fr2-Fr3 Protocol

1st round: Fr2kmn mix at 1000 nM, Jpool at 25 nM.

PCR protocol: 95 °C 1 min, 98 °C 1 min then 18 cycles of 95 °C 30 s, 50 °C 4 min, 70 °C 1 min.

2nd round: Fr3jkl mix at 1000 nM, Individual RAD at 30 nM, 5 µL 1/200 diluted PCR product.

PCR protocol: 95 °C 3 min then 25 cycles of 95 °C 30 s, 50 °C 2 min, 70 °C 90 s.

### 4.5. Fr2-Fr2 Protocol

1st round: Fr2kmn mix at 1000 nM, Jpool at 25 nM, Fr2Bmix at 1000 nM.

PCR protocol: 95 °C 1 min, 98 °C 1 min then 18 cycles of 95 °C 30 s, 50 °C 4 min, 70 °C 1 min.

2nd round: Fr2opq mix at 50 nM, Individual RAD at 30 nM, Fr2Bmix at 1000 nM, 5 µL 1/200 diluted PCR product.

PCR protocol: 95 °C 3 min then 25 cycles of 95 °C 30 s, 50 °C 2 min, 70 °C 90 s.

### 4.6. Fr1-Fr2 Protocol

1st round: Fr1 mix at 500 nM, Jpool at 25 nM.

PCR protocol: 95 °C 1 min, 98 °C 1 min then 20 cycles of 95 °C 30 s, 50 °C 3 min 30 s, 70 °C 90 s.

2nd round: Fr2opq mix at 1000 nM, Individual RAD at 30 nM, 5 µL 1/200 diluted PCR product.

PCR protocol: 95 °C 3 min then 25 cycles of 95 °C 30 s, 50 °C 2 min, 70 °C 90 s.

### 4.7. D Protocol

The D primers used were those described by Szczepanski et al. [13].

1st round: Dpool at 50 nM, Jpool at 25 nM, GladF at 50 nM, GladRev at 50 nM.

PCR protocol: 95 °C 1 min, 98 °C 1 min then 20 cycles of 95 °C 30 s, 60 °C 2 min 30 s, 70 °C 1 min.

2nd round: FAD5 at 50 nM, Individual RAD at 30 nM, 5 µL 1/200 diluted PCR product.

PCR protocol: 95 °C 3 min then 25 cycles of 95 °C 30 s, 70 °C 1 min.

Sequencing and bioinformatics analysis were performed by AGRF (Melbourne, Australia). Sequencing was performed on an Illumina MiSeq instrument (San Diego, CA 92122, USA) and produced 2 × 250 paired-end reads. After demultiplexing, bioinformatic analysis combined the paired ends and sorted the sequences by sample of origin. The criterion that a sequence was an immunoglobulin sequence was that it contained the 37-base conserved probe sequence with less than 10 mutations. The criterion that *immunoglobulin* sequences represented the same sequence was that, excluding the probe sequence, they differed by less than 5 mutations. Based upon these criteria, the sequences were merged and were finally ranked by frequency.

The criteria used to decide that an *immunoglobulin* sequence marked the myeloma clone were that the sequence comprised 5% or more of the IGH sequence reads, that it exceeded the frequency of the next common sequence by a factor of 5 or more, and that the total number of *IGH* reads was greater than 3000.

Mutations in the marker sequence were identified and enumerated between the end of the Fr2 site and the beginning of the J primer site or, when Fr2 information was not available, between the end of the Fr3 site and the beginning of the J primer site.

Patient-specific forward and reverse primers were synthesised with their sequence being based on the sequence of the identified *IGH* marker rearrangement. The Tm of the forward primer was 69.5–71.0 °C and the Tm of the reverse primer was 1–2 degrees higher. Each primer pair was tested in the PCR containing 5 ng of patient DNA/reaction and over a temperature gradient ranging from 68 °C to 75 °C to ensure that amplification at 72 °C was efficient. The number of target rearrangements added in the 5 ng of DNA present in the PCR was calculated from the mass of DNA/cell and the percentage of plasma cells observed in the marrow. The number of target rearrangements quantified by the PCR was calculated from the 2 Ct results obtained between 70 °C and 71 °C, which were on the plateau of the temperature gradient, by using 2.7 × 10^11^ as the number of amplified rearrangements at threshold, and by using 99.8% as the amplification efficiency of IGH primers [14]. The specificity of each primer pair was examined by incorporating the pair in 20 PCRs, each containing 1 µg of DNA which had been pooled from 5 healthy individuals.

## 5. Conclusions

The present study adds to our previous studies on the role of HAT-PCR in quantification of MRD in myeloma [10,11]. These studies have shown that HAT-PCR provides a sensitivity of 10^−6^, which is the same as that provided by NGS. However, in contrast to NGS, HAT-PCR is simple, cheap, quick and readily available. The high frequency of detection of a marker rearrangement by the present study therefore indicates that HAT-PCR can be used for measurement of MRD in the great majority of cases of myeloma.

## Figures and Tables

**Table 1:** Summary of results. This table indicates detection (+) or failure of detection (−) of a marker rearrangement. The results are arranged in increasing plasm cell frequency in the marrow sample.

Sample	% Plasma Cells	Primers					Summary
		D	Fr3:Fr3	Fr2:Fr3	Fr2:Fr2	Fr1:Fr2	
4-26	1.0	−	−	−	−	−	−
39-26	1.0	−	−	−	−	−	−
38-26	1.8	+	−	−	−	−	+
28-26	2.0	−	−	−	−	−	−
20-26	3.0	−	+	+	−	−	+
1-26	4.0	−	+	+	+	+	+
7-26	5.0	+	+	+	+	+	+
19-26	6.0	−	+	+	−	−	+
33-26	6.0	−	+	−	−	−	+
34-26	6.0	+	+	+	+	+	+
27-26	7.0	−	+	−	−	−	+
36-26	7.0	+	+	+	−	+	+
8-26	8.0	−	+	+	−	+	+
11-26	11.0	−	+	+	+	+	+
22-26	11.0	−	+	+	+	+	+
46-26	11.0	+	+	+	+	+	+
49-26	13.0	+	+	+	+	+	+
29-26	14.6	+	+	+	+	+	+
15-26	15.0	−	+	+	+	+	+
26-26	15.0	+	+	−	−	+	+
12-26	20.0	−	+	+	+	+	+
23-26	20.0	−	+	+	+	+	+
10-26	21.0	+	+	+	+	+	+
14-26	21.0	−	+	+	+	+	+
21-26	21.0	+	+	+	+	+	+
41-26	21.0	+	+	+	+	+	+
37-26	24.0	−	+	+	+	+	+
30-26	25.0	−	**−**	−	−	−	−
50-26	25.4	−	+	+	+	+	+
3-26	26.0	−	−	−	−	−	−
48-26	26.0	+	+	+	+	+	+
45-26	28.0	−	−	+	−	−	+
47-26	28.0	+	+	+	+	−	+
6-26	29.0	−	+	+	+	+	+
5-26	31.0	+	−	+	+	+	+
2-26	32.0	−	+	+	+	+	+
44-26	35.0	−	+	−	+	+	+
31-26	38.0	+	+	+	+	+	+
25-26	41.0	−	+	−	+	+	+
9-26	42.0	+	+	+	+	−	+
42-26	46.0	−	+	+	+	+	+
32-26	47.4	−	−	−	+	+	+
18-26	52.0	−	+	+	+	+	+
13-26	59.0	+	+	+	+	+	+
43-26	61.0	+	+	+	−	−	+
17-26	64.0	+	+	+	+	+	+
24-26	67.0	+	−	−	+	−	+
16-26	79.0	+	+	+	+	+	+
35-26	79.0	+	+	+	+	+	+
40-26	80.0	−	+	+	+	+	+

**Table 2:** Forward primers. The specific sequences designed to hybridise to the rearranged *immunoglobulin* gene are shown in lowercase and a portion or the complete sequence of the forward adapter is shown in uppercase. Fr3jkl, Fr2opq and FAD5 are primers for the second round of PCR. Fr3jkl, Fr2opq have at their 3′ends the specific sequences of Fr3ghi or Fr2kmn respectively.

Fr3mix	
Fr3g	GACGCTCTTCCGATCTnnnctgagagctgaggacacggctgtgtattactgt
Fr3h	GACGCTCTTCCGATCTnnnctgagagctgaggacacagccatgtattattgt
Fr3i	GACGCTCTTCCGATCTnnngtgacagccgtggacacggccgtgtattactgt
Fr3jkl	AATGATACGGCGACCACCGAGATCTACAC[index]ACACTCTTTCCCTACACGACGCTCTTCCGATCTFr3ghi spec seqs
Fr2mix	
Fr2k	tgggtccgccaggctccagggaaggggctggagtgg
Fr2m	tgggtccggcagcctccagggaagggcctggagtgg
Fr2n	tgggtgcgacaggcccctggacaagggcttgagtgg
Fr2opq	AATGATACGGCGACCACCGAGATCTACAC[index]ACACTCTTTCCCTACACGACGCTCTTCCGATCTFr2kmn spec seqs
Fr1mix	
VH1aFAD	AATGATACGGCGACCACCGAGATCTACACTCTTTCCCTACACGACGCTCTTCCGATCTnnngaagcctggggcctcagtgaaggtct
VH2-FAD	AATGATACGGCGACCACCGAGATCTACACTCTTTCCCTACACGACGCTCTTCCGATCTnnngtctggtcctacgctggtgaaaccc
VH3-FAD	AATGATACGGCGACCACCGAGATCTACACTCTTTCCCTACACGACGCTCTTCCGATCTnnnctggggggtccctgagactctcctg
VH4-FAD	AATGATACGGCGACCACCGAGATCTACACTCTTTCCCTACACGACGCTCTTCCGATCTnnncttcggagaccctgtccctcacctg
VH5-FAD	AATGATACGGCGACCACCGAGATCTACACTCTTTCCCTACACGACGCTCTTCCGATCTnnncggggagtctctgaagatctcctgt
VH6-FAD	AATGATACGGCGACCACCGAGATCTACACTCTTTCCCTACACGACGCTCTTCCGATCTnnntcgcagaccctctcactcacctgtg
Dpoolsz	
D1tagSz1	AATGATACGGCGACCACCGAGATCTACACTCTTTCCCTACACGACGCTCTTCCGATCTacccaggaggccccagagcaca
D1tagSz2	AATGATACGGCGACCACCGAGATCTACACTCTTTCCCTACACGACGCTCTTCCGATCTacccaggaggccccagagctca
D1tagSz3	AATGATACGGCGACCACCGAGATCTACACTCTTTCCCTACACGACGCTCTTCCGATCTatccaggaggccccagagcaca
D1tagSz4	AATGATACGGCGACCACCGAGATCTACACTCTTTCCCTACACGACGCTCTTCCGATCTatccaggaggccccagagctca
D2tagSz	AATGATACGGCGACCACCGAGATCTACACTCTTTCCCTACACGACGCTCTTCCGATCTcagcactgggctcagagtcctctc
D3tagSz1	AATGATACGGCGACCACCGAGATCTACACTCTTTCCCTACACGACGCTCTTCCGATCTcctcctcaggtcagccccggacat
D3tagSz2	AATGATACGGCGACCACCGAGATCTACACTCTTTCCCTACACGACGCTCTTCCGATCTcctcctcaggtcagccctggacat
D3tagSz3	AATGATACGGCGACCACCGAGATCTACACTCTTTCCCTACACGACGCTCTTCCGATCTcctcctccggtcagccccggacat
D3tagSz4	AATGATACGGCGACCACCGAGATCTACACTCTTTCCCTACACGACGCTCTTCCGATCTcctcctccggtcagccctggacat
D4tagSz1	AATGATACGGCGACCACCGAGATCTACACTCTTTCCCTACACGACGCTCTTCCGATCTcccaggacgcagcaccactgtcaa
D4tagSz2	AATGATACGGCGACCACCGAGATCTACACTCTTTCCCTACACGACGCTCTTCCGATCTcccaggacgcagcaccgctgtcaa
D5tagSz	AATGATACGGCGACCACCGAGATCTACACTCTTTCCCTACACGACGCTCTTCCGATCTacccagcctcctgctgaccagag
D6tagSz1	AATGATACGGCGACCACCGAGATCTACACTCTTTCCCTACACGACGCTCTTCCGATCTcaggccccccaaaaccagggat
D6tagSz2	AATGATACGGCGACCACCGAGATCTACACTCTTTCCCTACACGACGCTCTTCCGATCTcaggccccccaaaaccagggtt
D6tagSz3	AATGATACGGCGACCACCGAGATCTACACTCTTTCCCTACACGACGCTCTTCCGATCTcaggccccccaaaaccagtgat
D6tagSz4	AATGATACGGCGACCACCGAGATCTACACTCTTTCCCTACACGACGCTCTTCCGATCTcaggccccccaaaaccagtgtt
D6tagSz5	AATGATACGGCGACCACCGAGATCTACACTCTTTCCCTACACGACGCTCTTCCGATCTcaggccccccagaaccagggat
D6tagSz6	AATGATACGGCGACCACCGAGATCTACACTCTTTCCCTACACGACGCTCTTCCGATCTcaggccccccagaaccagggtt
D6tagSz7	AATGATACGGCGACCACCGAGATCTACACTCTTTCCCTACACGACGCTCTTCCGATCTcaggccccccagaaccagtgat
D6tagSz8	AATGATACGGCGACCACCGAGATCTACACTCTTTCCCTACACGACGCTCTTCCGATCTcaggccccccagaaccagtgtt
D7tagSz	AATGATACGGCGACCACCGAGATCTACACTCTTTCCCTACACGACGCTCTTCCGATCTgggctggggtctcccacgtgtttt
FAD5	AATGATACGGCGACCACCGAGATCTACAC[index]ACACTCTTTCCCTACACGACGCTCTTCCGATCT

**Table 3:** J primers and blockers. The specific sequences designed to hybridise to the rearranged *immunoglobulin* gene are shown in lowercase and portion of the adapter is shown in uppercase. The RAD primer was used in the second round with a different index for each sample. The blocker sequences incorporate “+” to indicate that the following base is locked and “/3Phos/” to indicate 3′ phosphorylation to inhibit extension.

Jpool	
AAMJ1bd	GTGACTGGAGTTCAGACGTGTGCTCTTCCGATCTctttgctgagcacctgtccccaagtctgaa
J1dupad	GTGACTGGAGTTCAGACGTGTGCTCTTCCGATCTcctcctgccgacctcctttgctga
J1j	GTGACTGGAGTTCAGACGTGTGCTCTTCCGATCTgccctcctgcttctcccatacaaaaacaca
setA J1d	GTGACTGGAGTTCAGACGTGTGCTCTTCCGATCTcctctgccctcctgcttctcccataca
setEJ2d	GTGACTGGAGTTCAGACGTGTGCTCTTCCGATCTgccccagggctaagtgacagca
J2k	GTGACTGGAGTTCAGACGTGTGCTCTTCCGATCTgggctctggcatgcagcccat
J2l	GTGACTGGAGTTCAGACGTGTGCTCTTCCGATCTcagggctaagtgacagcagggctct
Jset6 J3d	GTGACTGGAGTTCAGACGTGTGCTCTTCCGATCTacatggcccagcgcagaccaa
setA J3d	GTGACTGGAGTTCAGACGTGTGCTCTTCCGATCTcgtggtcccaaacagccggagaa
setC J3d	GTGACTGGAGTTCAGACGTGTGCTCTTCCGATCTccagttcccaaagaaaggccttctgctgaa
J4h	GTGACTGGAGTTCAGACGTGTGCTCTTCCGATCTctccggggctctcttggcagga
setA J4d	GTGACTGGAGTTCAGACGTGTGCTCTTCCGATCTctgttgcctcagggcatcctcctga
J4m	GTGACTGGAGTTCAGACGTGTGCTCTTCCGATCTgagcccttgcccctcgtctgtgt
J4dupad	GTGACTGGAGTTCAGACGTGTGCTCTTCCGATCTgcccttgcccctcgtctgtgt
J4j	GTGACTGGAGTTCAGACGTGTGCTCTTCCGATCTgggctcaggaggaaggagcatctgga
J5g	GTGACTGGAGTTCAGACGTGTGCTCTTCCGATCTcctgacctccaaaatgcctccaagactctga
J5dupad	GTGACTGGAGTTCAGACGTGTGCTCTTCCGATCTtccccagctttctttcctgacctccaa
setA J5d	GTGACTGGAGTTCAGACGTGTGCTCTTCCGATCTgaggacaggctgggttcccattcgaa
J5i	GTGACTGGAGTTCAGACGTGTGCTCTTCCGATCTgtggccggacttggggaggaca
setCa J5d	GTGACTGGAGTTCAGACGTGTGCTCTTCCGATCTctgccgacatctgtggccggactt
setA J6d	GTGACTGGAGTTCAGACGTGTGCTCTTCCGATCTgcccaggtcccctcggaacat
Jset6 J6d	GTGACTGGAGTTCAGACGTGTGCTCTTCCGATCTgaggaccaacctgcaatgctcaggaa
Jset5 J6d	GTGACTGGAGTTCAGACGTGTGCTCTTCCGATCTcggaaaatccacagaggctcccagatcc
setEJ6d	GTGACTGGAGTTCAGACGTGTGCTCTTCCGATCTagtcccattttccaaaggcatcggaa
setC J6q	GTGACTGGAGTTCAGACGTGTGCTCTTCCGATCTagcccccaggctcagttactccat
setC J6r	GTGACTGGAGTTCAGACGTGTGCTCTTCCGATCTttcaggcatctcgtccaaatgtggct
RAD	CAAGCAGAAGACGGCATACGAGAT[index]GTGACTGGAGTTCAGACGTGTGCTCTTCCGATCT
Blockers	
With Fr3-Fr3 protocol	
IA1bl3	TAC+TGT+ATG+TGA+GT+CA+CC+AG+GT+AA+GA+AG/3Phos/
IA2bl3	TAC+TGT+GCGAAA+GAC+ACA+GTG+AGG+GGA+AGT/3Phos/
With Fr2-Fr2 protocol	
Fr2B1	GAG+TGG+AGTGGG+GAGAGCCAGAGA+GGAATG+GGG+ACA/3Phos/
Fr2B2	GAG+TGG+AGTGGG+GAGAGCAGTAGC+AGGTGA+GGC+CA/3Phos/
Fr2B3	GAG+TGG+AGGGGG+CAGAGGGACCCC+CAGGCA+GGG+CCG/3Phos/
Fr2B4	GTG+GGG+GGAGGG+AAGGGCCCA+GAGCCC+GAG+CTAC/3Phos/
Fr2B5	GAG+TGG+GGCCAA+GTCCTGGAAAATGAGGTT+ACTTCT+CCA+AG/3Phos/
Fr2B6	GAG+TGC+CACTCC+GTCCATCCAGACTTC+AAA TGG+ACC+CAC/3Phos/
Fr2B7	GAG+TGG+GGGCTG+GGCATCGGAGAGGC+ACGGCC+TCC+TGC/3Phos/
With D protocol	
GladF	CAGTGATTGGCAGCTCTACAAAAACCATGCT
GladRev	CCTGAGCCAGGGGCTACAGAAA

## Data Availability

The original contributions presented in this study are included in the article material. Further inquiries can be directed to the corresponding author.
